# A conceptual review on reconstructive peri‐implantitis therapy: Challenges and opportunities

**DOI:** 10.1002/cre2.788

**Published:** 2023-09-21

**Authors:** Hsun‐Liang Chan, Amanda Rodriguez Betancourt, Chun Ching Liu, Yi‐Chen Chiang, Patrick R. Schmidlin

**Affiliations:** ^1^ Department of Periodontics and Oral Medicine The University of Michigan School of Dentistry Ann Arbor Michigan USA; ^2^ Center of Dental Medicine, Division of Periodontology and Peri‐implant diseases, Clinic of Conservative and Preventive Dentistry University of Zurich Zurich Switzerland

**Keywords:** microsurgery, peri‐implantitis, ultrasonography, wound healing

## Abstract

**Objectives:**

The current strategies to reconstruct lost peri‐implant tissues due to the disease have been largely unpredictable. The aim of this conceptual review is to discuss relevant biological and biomechanical challenges of applying reconstructive means to treat peri‐implantitis. Additionally, opportunities to improve treatment predictability are presented.

**Material and Methods:**

A narrative review was conducted to fulfill the aim.

**Results:**

The four interrelated negative conditions hampering effective reconstruction are: inferior tissue perfusion, unfavorable bone topography, ineffective surface treatment, and unstable wound. First, peri‐implant tissues resemble scars with reduced cellularity and vascularity, coupled with the absence of the periodontal ligament plexuses and the avascular implant and biomaterials, maintaining primary closure is a challenge, which is critical for regeneration. Second, defect morphology and bone topography surrounding implants determine the reconstructive potential. Unfortunately, noncontained defects are frequently encountered, with a combination of suprabony (horizontal bone loss) and infrabony (vertical usually involving circumferential bone loss) defects. Third, current attempts for implant surface decontamination are insufficient due to inaccessible macrostructure and rough surfaces in the micro‐scale. Histologic evaluation has shown bacteria aggregation and calcified deposits around implants. Lastly, wound stability is difficult to achieve due to inherent soft tissue biomechanical quality and quantity deficiencies and mobile bone particulates. Opportunities to tackle the abovementioned challenges include the use of novel imaging technologies, such as high‐frequency dental ultrasound and laser speckle imaging to evaluate tissue perfusion, soft tissue quality/quantity, and bone topography pre‐surgically. The use of the operating microscope could allow better visualization and removal of etiologic factors. Strategies to improve soft tissue quality may include preoperative control of soft tissue inflammation and the potential use of biologics. Methods such as fixation to stabilize the biomaterials could be beneficial.

**Conclusions:**

A more nuanced understanding of the current challenges and opportunities can lead to more effective preoperative and postoperative care protocols, ultimately improving the success rate of reconstructive procedures.

## 1 INTRODUCTION

Oral implants are a widespread solution for restoring oral function and esthetics, with a growing number of patients receiving at least one implant; however, while the overall outcomes are promising, a significant subset of implants, estimated to be between 10% and 20%, experience peri‐implantitis, a destructive inflammatory process affecting both soft and hard tissues around dental implants (Alghamdi & Jansen, 2020; Derks et al., 2016). Peri‐implant mucositis, characterized by soft tissue inflammation without pathologic bone loss, and peri‐implantitis, characterized by both soft and hard tissue manifestations, are the primary two types of inflammatory peri‐implant diseases classified by recent research (Derks et al., 2016; Renvert et al., 2018).

The diagnosis of peri‐implantitis is based on various criteria, including the depth of probing exceeding 6 mm, bleeding on probing, and/or the presence of suppuration/pus. Without a baseline radiograph for comparison, bone loss of more than 3 mm indicates peri‐implantitis, compared to 2 mm when a baseline radiograph is available (Heitz‐Mayfield & Salvi, 2018; Schwarz et al., 2018). The prevalence of peri‐implantitis varies in the literature, primarily due to differences in population and disease definition (Derks & Tomasi, 2015). Peri‐implantitis is believed to be caused by bacterial pathogens in susceptible individuals, leading to the loss of supporting bone and eventually, the implant, causing a significant financial burden and affecting patients' welfare (Schwendicke et al., 2015). Similar to periodontitis, peri‐implantitis is still primarily treated following periodontal surgical strategies to halt disease progression and rescue the implant. While nonsurgical therapy in combination with proper oral hygiene reinforcement remains a basic standard of care and the first step, surgical measures are required in advanced cases since nonsurgical protocols with an adjunctive or alternative failed to demonstrate efficacy in resolving the disease (Ramanauskaite et al., 2021). From a surgical perspective, regeneration is desirable as it has the potential to restore the function and architecture of lost tissues. The literature reports the efficacy of reconstructive procedures, with mixed results. Systematic reviews show that the average bone gain is 2−3 mm; however, there is still a good portion of cases that are not resolved. Wide variations in reported results may be attributed to the heterogeneity in the severity and variation of the disease, selection of surgical techniques and materials, surface decontamination methods, surgeons' skills, and other factors (Tomasi et al., 2019).

There is a need to identify the obstacles to effectively and predictably regenerate peri‐implant tissues from biologic and biomechanical viewpoints to develop meaningful research strategies and evidence‐based treatment protocols (Solderer & Schmidlin, 2020). While most work in this field focuses on biomaterials and related surgical topics, the primary aim of this manuscript is to discuss relevant biological and biomechanical challenges of treating peri‐implantitis based on the surgical biological regeneration principles. Strategies to overcome these challenges are suggested for future validation with research and clinical evaluations and modifications in the upcoming sections.

## 2 CHALLENGES OF RECONSTRUCTING PERI‐IMPLANT TISSUES

Four challenges are summarized in Table 1 and will be discussed below.

**Table 1:** Descriptive challenges on peri‐implant reconstructive procedures.

Challenges in category	Negative factors
Tissue perfusion	Scar‐like tissues at baseline Interrupted nutritional diffusion from bone Avascular implant underneath Compromised microvasculature after tissue releasing
Bony topography	Higher incidence of non‐contained defect Reduced progenitor cell resource
Implant surface decontamination	Calcified deposits on implant surface Inaccessible macrostructure Rough surfaces in micro‐scale
Biomechanical wound stability	Inflamed wound edge with weakened tensile strength Limited keratinized mucosa Soft tissue flap recoil Unstable biomaterials A challenge to obtain primary wound closure

## 3 POSTOPERATIVE TISSUE PERFUSION

**Figure 1:**
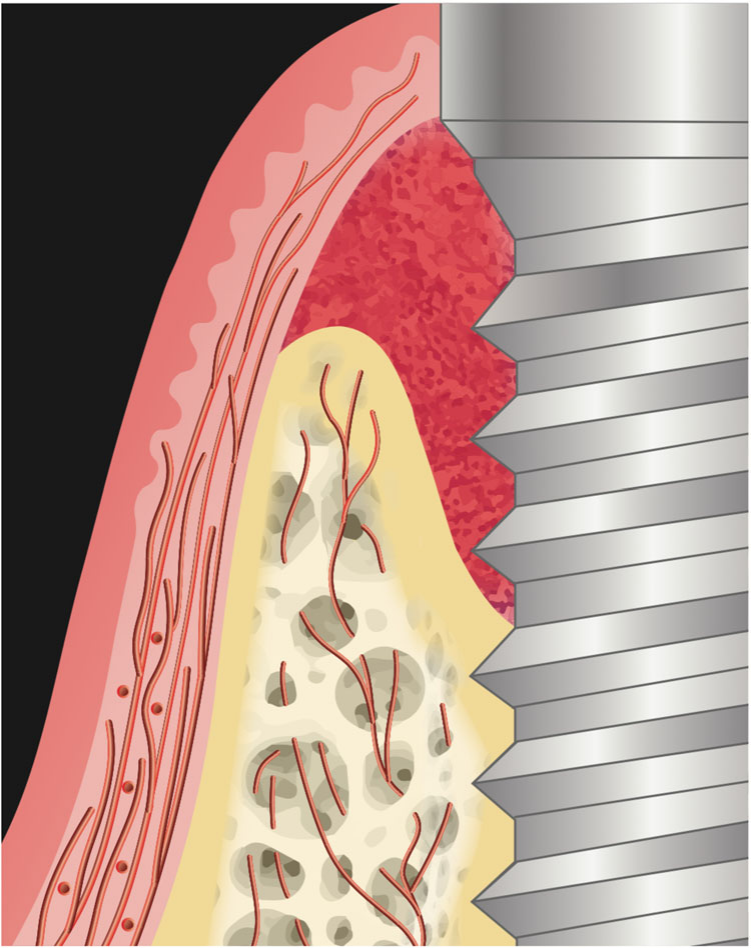
Tissue perfusion in the peri‐implant tissues. Arterioles have an apico‐coronal direction and they can be intraosseous, supraperiosteal, and in the peri‐implant soft tissue. The vessel dimension and density decrease when they pass to the mucogingival junction. They have a parallel orientation with the implant. Only small anastomoses of the arterioles reach the mucosal margin and the peri‐implant crestal bone.

One of the most common postoperative complications of oral tissue reconstruction is soft tissue dehiscence at the wound edge, resulting in sustained inflammation, disrupted granulation tissue formation, epithelial down growth, and loss of biomaterials (Naenni et al., 2019). These negative events eventually increase the risk of converting to unfavorable clinical outcomes. Insufficient tissue perfusion at the wound edges might have partially accounted for wound opening (Fugazzotto, 1999). The majority of the periodontal/peri‐implant tissue perfusion arises from the supra‐periosteal plexuses (SPP) at the base of the flap (Rodriguez Betancourt & Chan, 2023). The latter reduce in number and diameter as they travel from the lining mucosa to the attached mucosa (Figure 1). In other words, the attached mucosa/keratinized mucosa is less perfused and is vulnerable to ischemia and necrosis under normal conditions (Retzepi et al., 2007). However, from biomechanical point of view, a minimal amount of this type of tissue is needed to facilitate suturing and wound closure. Specifically for peri‐implant tissues, preclinical studies have shown they are essentially scar tissues with less vascularization and cellularity, compared to the periodontium (Sculean et al., 2014). Therefore, the baseline peri‐implant tissue perfusion is already at its disadvantage. After flap reflection, the microvasculature communicating between the hard and soft tissue interface is disrupted (Hagenaars et al., 2004; Mörmann & Ciancio, 1977; Nobuto et al., 2003). During the first few days of healing, nutrient diffusion from the bone to the soft tissue also serves the role of sustaining the flap vitality. With placed biomaterials in between the residual bone and soft tissue flap and the presence of an avascular implant underneath the flap, this diffusion is compromised. What might further compromise the tissue perfusion is the tissue‐releasing steps that are performed to allow for coronal advancement of the flap (Shaikh et al., 2021). These releasing steps, either the periosteal scoring, the pouch technique, or vertical incisions, have the potential to disrupt the microvasculature (Rodriguez Betancourt & Chan, 2023). These aspects might contribute to insufficient tissue perfusion at the wound edge after the reconstructive procedures that result in wound dehiscence and unpredictable outcomes (Table 1).

## 4 BONE DEFECT MORPHOLOGY

**Figure 2:**
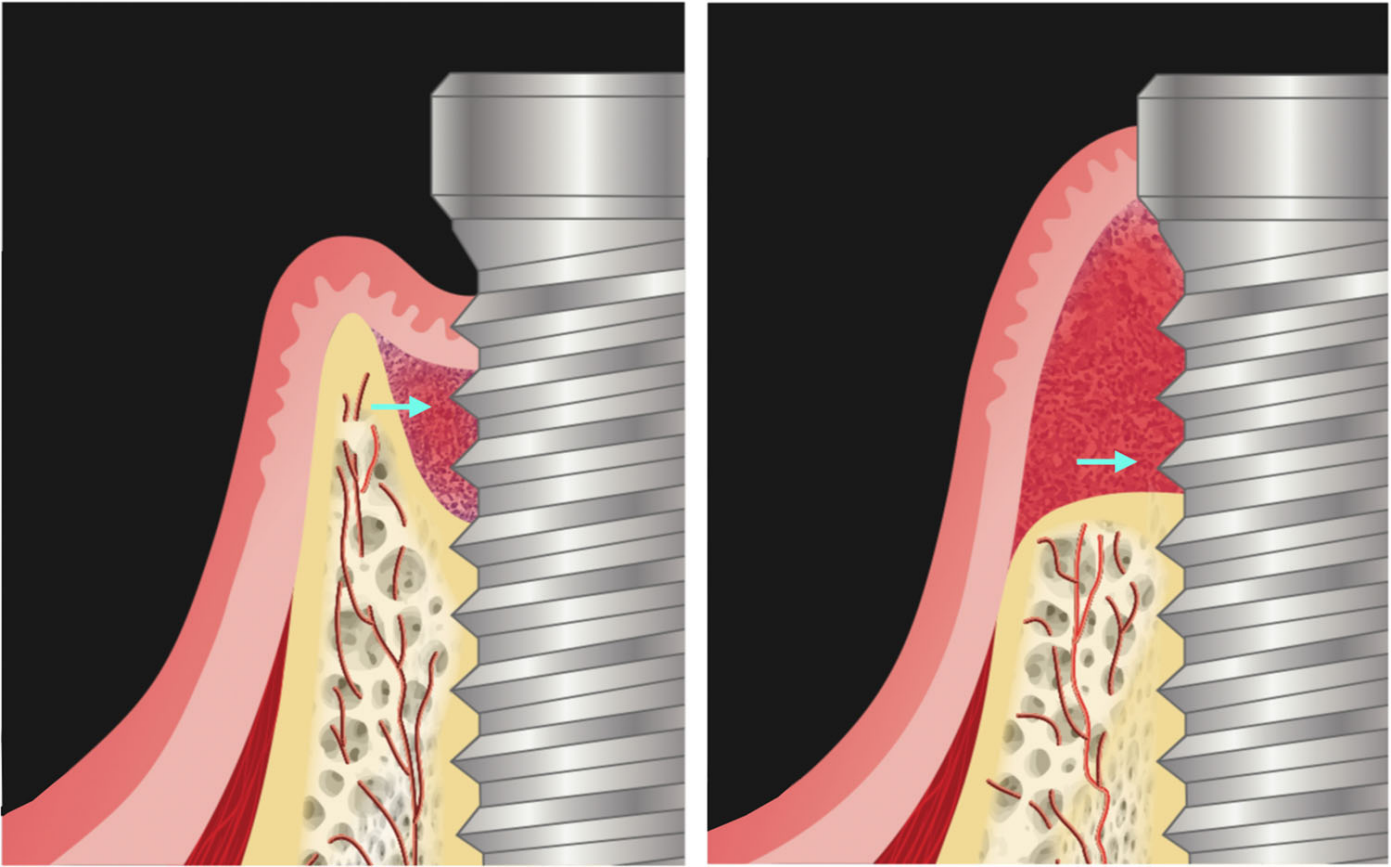
Bony topography surrounding infected implants. The left image shows a vertical defect with an infrabony component and a small suprabony component (a cyan arrow shows the space between the implant platform and the bone crest). The right image shows a horizontal defect without an infrabony component (a cyan arrow shows the suprabony defect above the bone crest).

Bony topography surrounding infected implants is a basic factor, which determines the intrinsic conducive potential leading to defect regeneration (Schwarz et al., 2010). Such defects typically comprise suprabony and infrabony components (Figure 2). With regard to the infrabony component, bone loss patterns usually involve circumferential loss, affecting both interproximal and facial bones. In some cases, the palatal/lingual bone is also lost. These defects tend to be wide, ranging from approximately 1.5−2 mm. The creation of such dimensions results from the body's attempt to isolate the source of infection at the expense of losing peri‐implant bone volume (Monje et al., 2019). Regenerating these types of defects typically requires a combination of vertical and horizontal bone augmentation, which has been challenging even for augmenting the alveolar ridge or around pristine implants. Especially, the presence of the suprabony bony component makes bone reconstruction even more challenging (Jepsen et al., 2019). Currently, regenerating the bone loss coronal to the interproximal crestal bone is not possible (Giannobile & Somerman, 2003; Jin et al., 2003). Thus, the regeneration potential of such defects is determined by the relative spatial relationship between the implant platform and the interproximal crestal bone. Complete recovery of the bony defect around the implant may be only possible if the interproximal bone remains coronal to the platform; otherwise, the suprabony component of the implant is not expected to be covered by bone even after a successful procedure.

From a biological concept of peri‐implant osseous formation, reconstruction requires the presence of bone‐forming/progenitor cells in the surrounding vicinity of the defect, since progenitor cells can only derive from the remaining bone tissues (Bosshardt et al., 2017). The distance between the residual bone and the implant, along with missing bone walls, may limit the source of bone‐forming cells critical for bone reconstruction in peri‐implant defects. Therefore, defects with missing bone walls and wide defects present a significant challenge for predictable bone reconstruction, which is usually the case in clinical scenarios.

### 4.1 Defect degranulation and decontamination

A crucial step in peri‐implant treatment lays in adequate defect cleaning. The focus is put on the degranulation of the soft tissue‐filled defect on one side and the decontamination of the affected exposed implant on the other side. The implications and consequences of removing granulation tissue on the healing process have been evaluated in various ways over time. As a typical example, granulation tissue is formed after tooth extraction and bears the notable and important potential to differentiate into autologous bone and filling up even empty defects (Trombelli et al., 2008). While it is obvious from a practical point of view that granulation tissues need to be removed, especially whenever defects are compensated with fillers, complete removal of it has also been questioned since multipotent progenitor stem cells can be even identified in infected granulation tissues (Ronay et al., 2013). Therefore, the common practice of removing all granulation tissue during bone surgery may also result in the removal of vital multipotent stem cells that could lead to favored tissue healing if retained. Studies have also assessed nonsurgical debridement and local detoxification leaving deliberately granulation tissue in the peri‐implant pockets (Crespi et al., 2019), and periodontal tissues (Crespi, Capparé, Bollero, et al., 2016; Crespi, Capparé, Gastaldi, et al., 2016; Ronay et al., 2013) with promising results, however further longitudinal studies are required.

**Figure 3:**
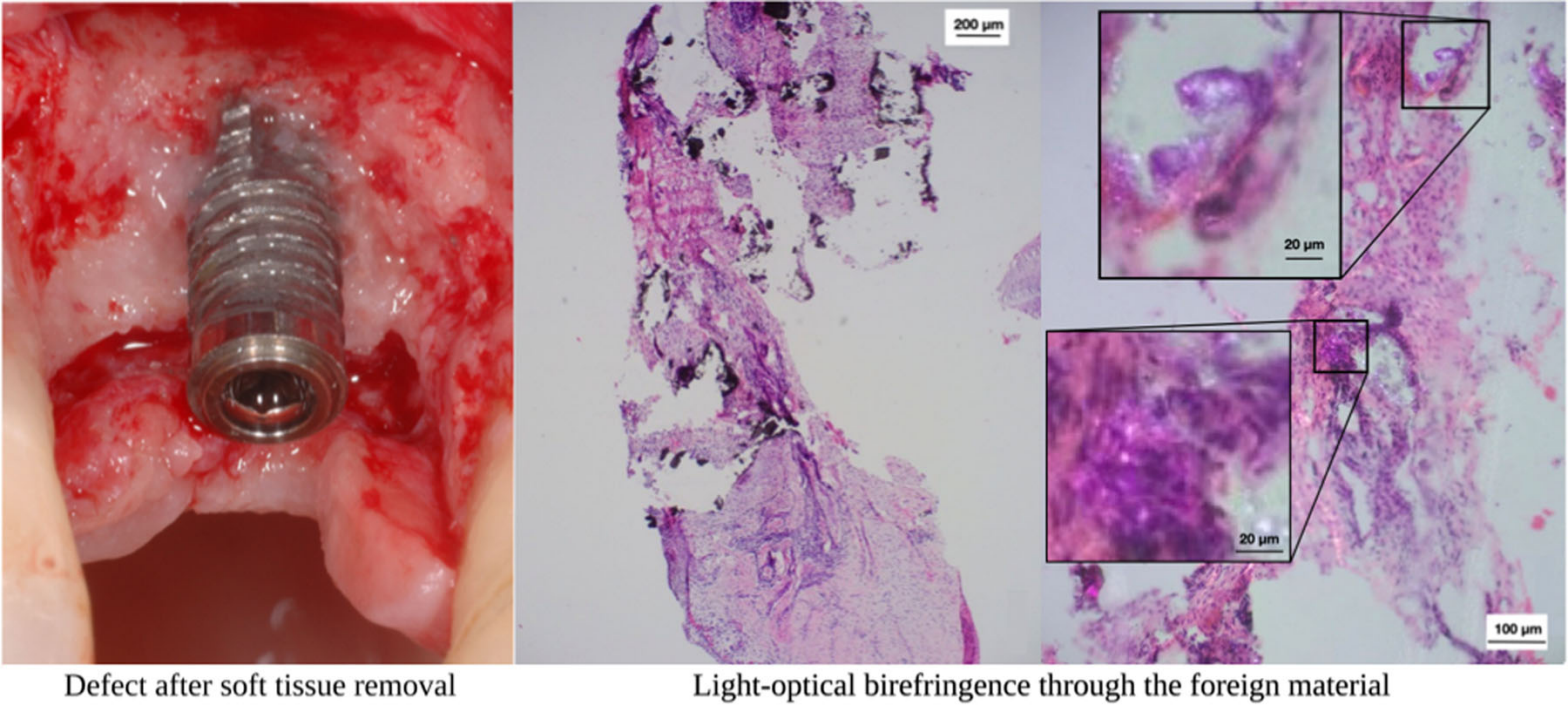
Clinical situation of a peri‐implant defect after degranulation and cleaning (left), along with a biopsy fragment overview and histology (middle). The right image focuses on a detailed view of foreign material using light‐optical birefringence.

**Figure 4:**
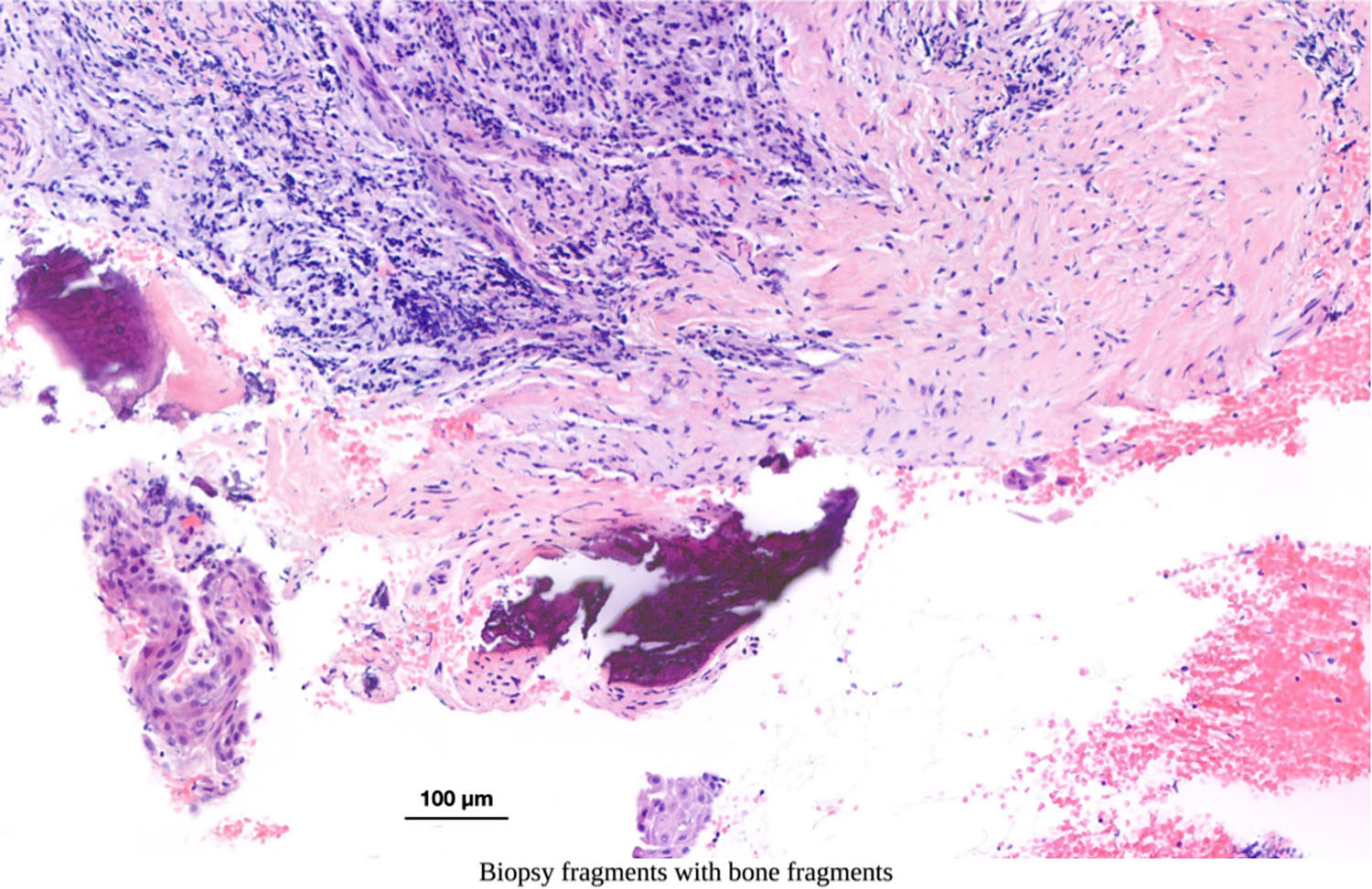
Histologic image capturing an excised sample from another case, revealing the presence of bone fragments (dark staining).

**Figure 5:**
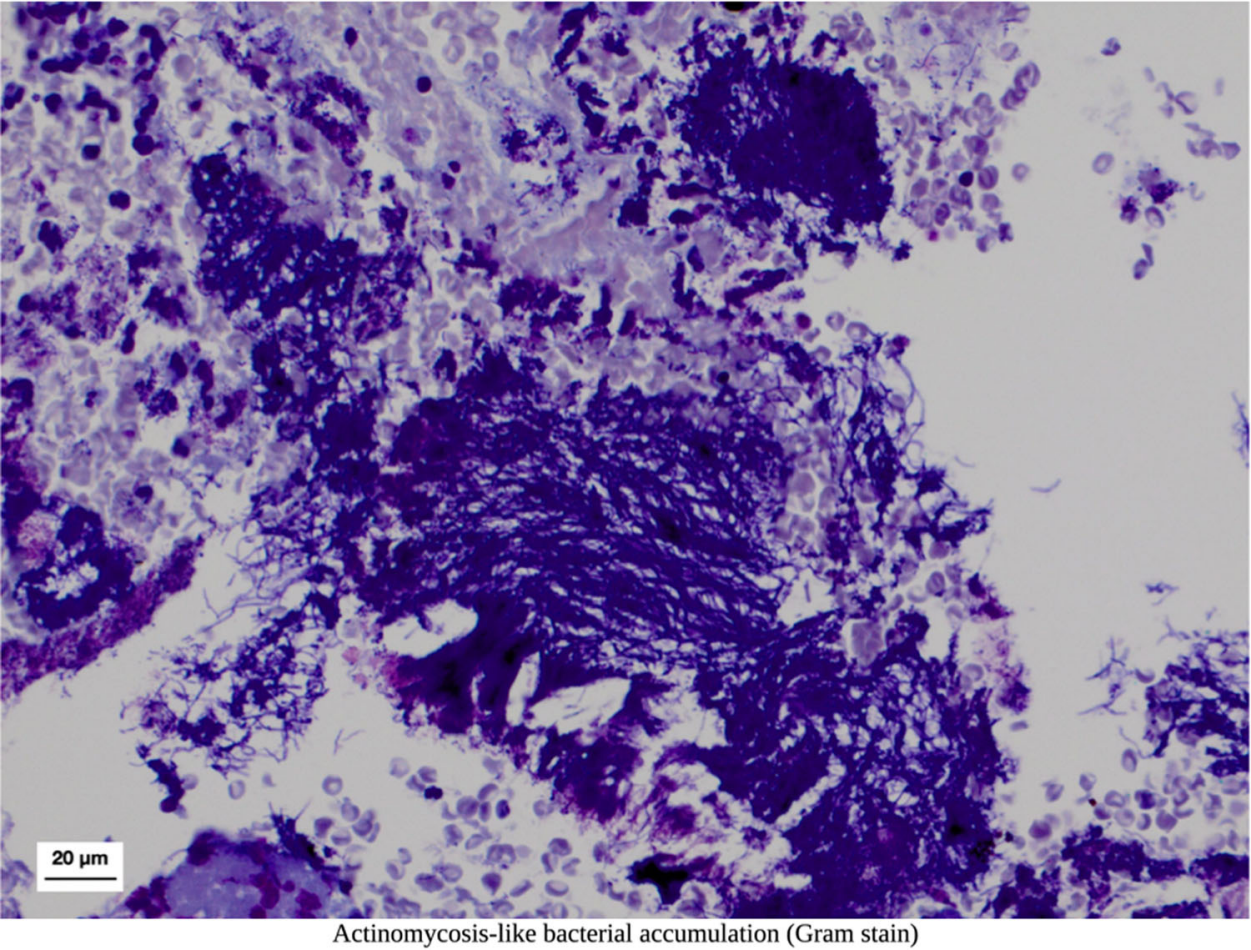
Histological example demonstrating the accumulation and aggregation of bacteria resembling actinomycosis.

A closer look at histology taken from excised material from pathologically altered peri‐implant soft tissues highlights the crucial role of histological analysis, understanding, and diagnosing peri‐implant defects. Figures 3, 4, 5 depict fragmentary overviews and histologic highlighting different foreign bodies and bacterial aggregates, which themselves showcase the potential role of histological analysis in diagnosing peri‐implant defects, underscoring the need for accurate assessment of tissue composition and structure to determine the severity and nature of the defect, enabling personalized treatment planning and intervention. In this context, one must therefore keep in mind that leaving granulation tissues behind potentially increases the risk of leaving nonvital foreign material with pathogenic character and potential, as well as leaving infectious material that challenges the immune system and complicates healing.

In addition, remaining granulation tissues also interfere with the proper dental implant surface debridement, inspection, and control; the access to the implant surface is restricted in addition by the location in the oral cavity, the bony wall configuration (width and depth), and implant supra‐structure, which also limit the ability to effectively reach and decontaminate the implant surface. System‐inherent thread designs of modern implants create crevices that are difficult, if not impossible, to reach with currently available armamentarium. Even under optimal in vitro conditions, instruments display unprocessed areas depending on the implant designs; in addition, titanium remnants can be found in the surrounding tissues, especially after treatment with mechanically more aggressive instruments (Fischer et al., 2023), which then leads to the controversially discussed and anticipated unwanted biological sequelae (Ivanovski et al., 2022).

Beside inevitable soft deposits, that is, biofilms, a second challenge is the presence of calcified deposits, which may adhere to the implant surface (Figuero et al., 2014). Simple chemical treatment or mechanical blasting with glycine or erythritol is insufficient to remove such calcified deposits. The inability to visualize these concerns, including vital biofilms with our naked eyes or loupes represents a significant challenge. Bleeding may additionally interfere with proper visual control. Without proper access/visual, effective decontamination is like finding a needle in a haystack.

In summary, effective surface decontamination remains a major obstacle for reconstructive peri‐implant tissues resulting from peri‐implantitis. But even after ideal defect degranulation and decontamination, re‐osseointegration may not be an attainable and realistic goal; rather the aim is to recreate a biocompatible implant surface that allows for inflammation resolution, bone reapproximation, and the elimination or reduction of peri‐implant pockets to a maintainable status.

## 5 POSTOPERATIVE WOUND STABILITY

Stable tissue support is crucial to facilitate the reconstruction of periodontal or peri‐implant defects (Susin et al., 2015). There are essential biological principles and conditions that can unleash the innate potential of the tissues to attain optimal reconstruction, especially when flaps are mobilized coronally and maintained in a new position for adequate wound coverage, while biomaterials must be immobilized for bone conduction and maturation. The basic principles encompass the biological trinity of space provision, wound stability, and optimized conditions for primary intention healing. Therefore, the final success of reconstructive procedures, especially in the critical peri‐implant wound system, lays in long‐term stable, vital, and infection‐free soft tissue conditions after surgery.

Clinicians are aware of multiple factors, which may hamper the desired tissue stability in an already critical and fragile system (Burkhardt & Lang, 2014). We know that sustained and non‐resolving inflammation results in reduced collagen content and inferior tissue quality, leading to weakened tissue tensile strength. Secondly, limited keratinized mucosa width, particularly following prior tissue destruction due to periodontitis, is associated with a higher incidence of wound dehiscence after reconstructive procedures. Thirdly, soft tissue flaps tend to return to their original position due to viscoelastic properties, muscle pulls, and postoperative swelling (Burkhardt & Lang, 2010). Finally, biomaterials are typically mobile unless fixation methods are employed, which can lead to soft tissue invagination and decreased opportunity for consolidation and maturation (Gallo et al., 2022).

The interrelationships between residual bone topography, quality/quantity of soft tissue flaps, macro‐ and microstructures of implant surfaces, and mechanical properties of biomaterials placed are critical in determining the success of reconstructive procedures around infected implants. These factors are interconnected and must be considered together, including the size of suprabony defects that may be related to the amount of coronal flap advancement needed for primary closure, which in turn affects the blood perfusion and biomechanical properties of the soft tissue. Balancing conflicting factors is also essential, as extensive flap release can compromise tissue perfusion. The critical and profound understanding of the influencing factors that negatively affect wound stability is crucial in the development of successful reconstructive procedures for peri‐implantitis. A comprehensive approach that considers all the interrelated factors is necessary to achieve predictable outcomes.

## 6 DISCUSSION

**Figure 6:**
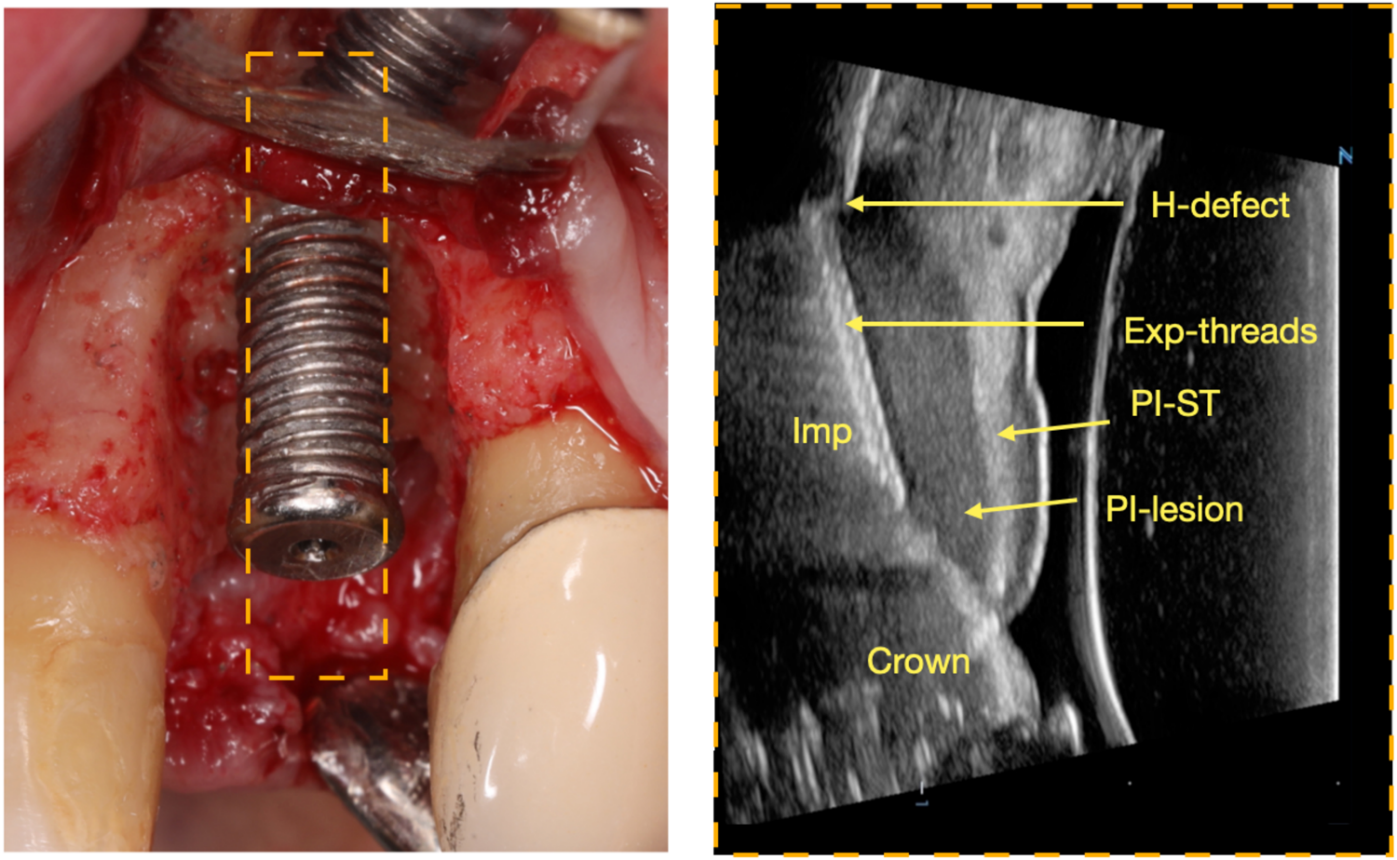
Preoperative ultrasound image (Right) before crown removal that can be used for treatment planning regarding the defect type, amount of facial bone loss, and soft tissue quality. In this case, a relative horizonal facial defect (H‐defect) was found, with exposed implant threads (Exp‐threads), peri‐implant lesion (PI‐lesion) and thick peri‐implant tissue (PI‐ST). The image was confirmed during open surgery (Left).

Challenges often provide the impetus for innovation and opportunities. The identification of these challenges creates a pathway for the formulation of strategic solutions. This is particularly relevant in the context of enhancing the predictability of reconstructive procedures for treating peri‐implantitis. The current overview contributes to this evolving area of research by proposing several aspects and strategies that can inform both research development and clinical practice. These strategies are detailed in Table 2. A significant area of focus relates to tissue perfusion, a crucial factor in the success of reconstructive procedures. High‐frequency dental ultrasound and laser speckle imaging technologies can be employed to precisely locate the peri‐implant lesion and quantify the baseline tissue perfusion (Barootchi et al., 2022; Chan & Kripfgans, 2020). The real‐time, spatially‐resolved data generated by these technologies provide invaluable information in the future that aids in flap design and decision‐making regarding the use of biologics, thereby enhancing preoperative planning (see Figure 6 and Supporting Information: Video 1).

**Table 2:** Proposed strategies during the preoperative and intra‐surgical treatment phases.

Challenges in category	Proposed strategies	
	During diagnosis/planning phase	During treatment phase
Tissue perfusion	Identify SPP locations and quantify tissue perfusion	Minimize trauma to microcirculation Controlled flap releasing Use of biologics, for example, rh‐PDGF‐bb
Bony topography	Evaluate bone topography	Aim for regenerating infrabony defects Use of autogenous bone Use of biologics, e.g., BMP‐2 and osteoinductive bone grafts
Implant surface decontamination	Aware of other disease causes	Apply high magnification, for example, operating microscope Remove suprastructure for better access Design miniature‐sized debridement tips
Biomechanical wound stability	Soft tissue quality/quantity evaluation Consider removal of suprastructure for submerged approach to achieve primary wound closure	Preoperative control of soft tissue inflammation Suture/fixate biomaterials Control tissue swelling

Abbreviation: rh: PDGF‐bb, recombinant human Platelet‐Derived Growth Factor‐BB.

During surgical intervention, careful attention should therefore be paid to preserving tissue integrity and minimizing trauma to the microcirculation. A meticulous approach toward the sharp dissection of the flap from inflammatory granulomatous tissues can minimize the risk of flap tears. Furthermore, maintaining tissue hydration is critical to ensuring its viability and optimal healing post‐surgery. Advances in microsurgical techniques, such as the visual and controlled release of flaps under high magnification, offer considerable benefits. In addition, the judicious application of biologics such as recombinant human Platelet‐Derived Growth Factor‐BB (rh‐PDGF‐bb) may stimulate angiogenesis, thereby enhancing transient vascularization and promoting tissue reconstruction as highlighted since years (Nevins et al., 2005).

**Figure 7:**
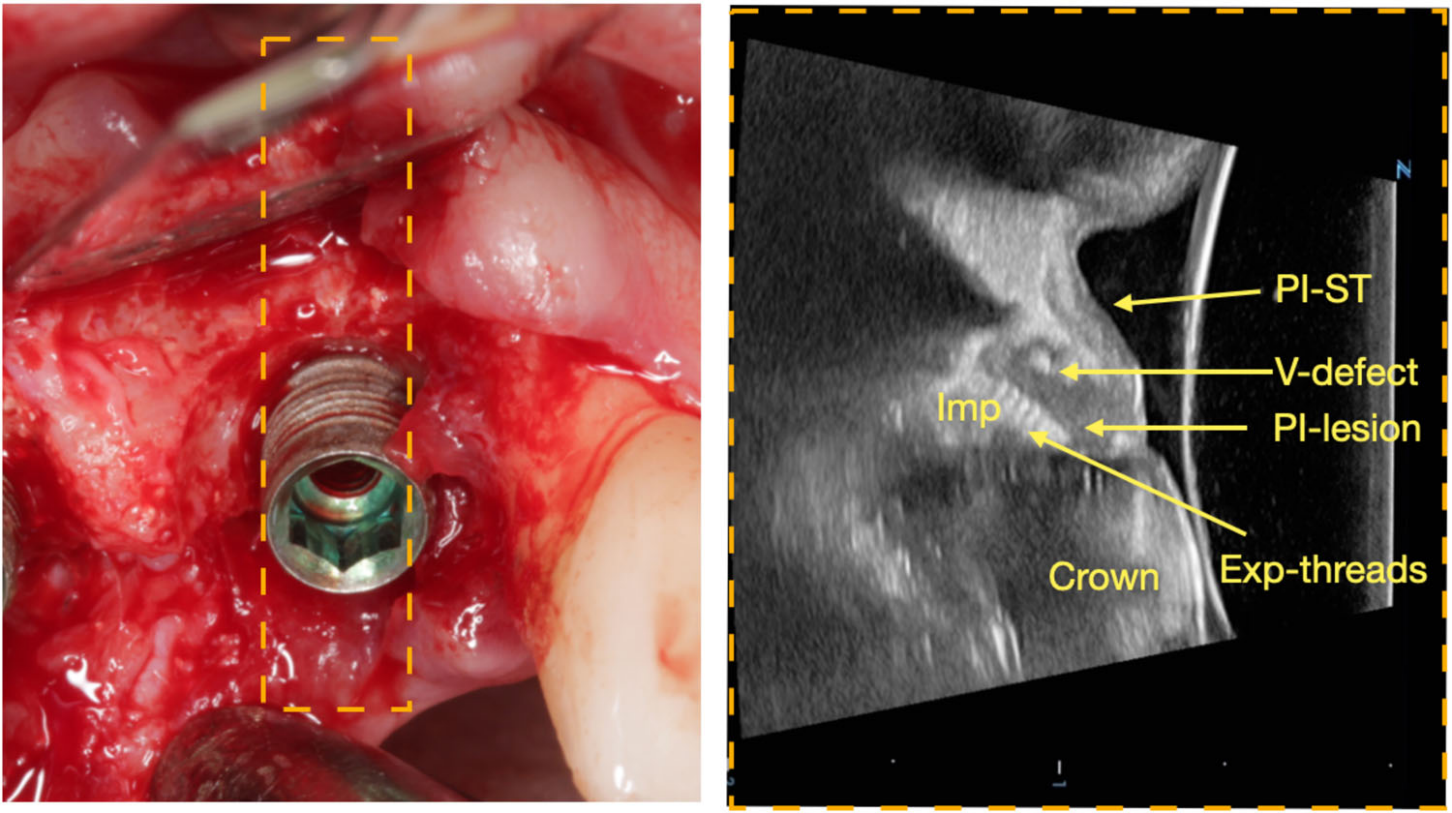
Preoperative ultrasound image (Right) before crown removal that can be used for surgical planning. Facial bone has a small infrabony component, more amenable for regeneration. Clinical intra‐surgical photo confirmed the vertical defect on the buccal wall (Left). Exp‐threads, Exposed implant threads; PI‐lesion, peri‐implant lesion; PI‐soft tissue, peri‐implant soft tissue.

The careful assessment and optimization of bony topography is another critical factor in reconstructive procedures. Technological advancements, such as cone‐beam computed topography (CBCT) and again high‐frequency dental ultrasound, provide high‐resolution images that can be used to determine the feasibility of reconstruction (Figures 6 and 7) (Patel et al., 2015). Further, the strategic use of advanced barrier membranes with long‐term stability and osteoconductive potential as well as respective bone grafts with higher intrinsic osteoinductive potential, along with human recombinant bone morphogenetic proteins type‐2 (rhBMP‐2) or other biologicals, can significantly augment the reconstructive potential of the site (James et al., 2016).

Noteworthy, surface decontamination remains paramount to the success of any implant reconstruction. Factors extending beyond dental plaque, including excessive cement and implant platform fracture, should be evaluated preoperatively. Pathogenic alterations of the granulation tissues should also be considered. Facilitation of access through the removal of the implant superstructure, coupled with the use of an operating microscope with high magnification (~X30) and coaxial illumination, can enhance the identification and removal of dental plaque and calcified deposits on the implant surface (see Supporting Information: Video 2). Miniature‐sized ultrasonic tips, designed for navigating through the peaks and valleys of the implant, may further optimize this process.

In addition, preoperative control of soft tissue inflammation can significantly improve tissue quality and wound stability, thereby creating a favorable environment for reconstruction. Immobilization of bone particulates and the membrane using fixation pins/screws and sutures can provide mechanical stability and prevent micromotion, which is often detrimental to bone healing (see Video 3). Ensuring minimal surgical trauma and adopting targeted flap release can further reduce tissue swelling, which can potentially compromise postoperative healing and the success of the reconstructive procedure.

Additional benefits of suprastructure removal are to reduce inflammation and to enhance wound closure in the submerged approach (Wen et al., 2022). The submerged approach aims to obtain primary wound closure for undisturbed wound healing favoring the biological principles of bone regeneration (Daugela et al., 2016; de Tapia et al., 2019; Galarraga‐Vinueza et al., 2020; Jepsen et al., 2016; Wang & Boyapati, 2006). In addition, the prosthesis may be a contributing factor in the initiation or development of the disease. Thus, refabrication or adjustment could assist in sustaining the treatment outcomes (Katafuchi et al., 2018; Wen et al., 2022). Positive outcomes can be seen using this approach with radiographic bone gain and pocket depth reductions (Isler et al., 2018; Monje et al., 2019; Roos‐Jansåker et al., 2007a, 2007b; Wen et al., 2022).

While these strategies are grounded in established scientific principles, they necessitate rigorous scientific research for their validation. The inconsistent use of devices offering higher magnification, such as the operating microscope, might contribute to the variability in reconstructive treatment outcomes. Compared to traditional dental loupes offering magnification of around X3.5, the operating microscope provides a maximal magnification of ~X30 (Bud et al., 2021). This superior level of magnification can enable clinicians to discern intricate details, thereby enhancing precision and facilitating comprehensive decontamination and reconstructive procedures.

Additional research is also needed to evaluate the use and efficacy of different biologics. While preliminary findings regarding the use of a plethora of growth factors in stimulating angiogenesis and osteogenesis are promising, further empirical investigations should yield more concrete evidence regarding their clinical effectiveness, optimal dosage, and potential side effects, especially in peri‐implant defects (James et al., 2016; Nevins et al., 2005). Moreover, the design and application of ultrasonic tips for effective navigation through implant structures represent another area ripe for further scientific exploration. The development of efficient, miniature‐sized tips could revolutionize the process of implant surface decontamination, thereby enhancing the outcomes of reconstructive procedures.

## 7 CONCLUSION

In summary, the interplay between soft tissue inflammation control, flap design, and wound stability also warrants further investigation. A more nuanced understanding of these factors can guide the development of more effective preoperative and postoperative care protocols, ultimately improving the success rate of reconstructive procedures.

Finally, the proposed aspects and possible strategies underpin the importance of a comprehensive and integrative approach toward effective peri‐implantitis treatment. Only addressing tissue perfusion, bony topography, surface decontamination, and soft tissue inflammation holistically can significantly enhance the predictability and effectiveness of reconstructive procedures. Future scientific studies should, therefore, aim to examine these strategies not just in isolation but also in combination to provide a more robust and nuanced understanding of the optimal treatment regimen for peri‐implantitis.

## AUTHORS CONTRIBUTIONS

*Conceptualization*: Hsun‐Liang Chan, Chun Ching Liu and Patrick R. Schmidlin. *Data curation*: Amanda Rodriguez Betancourt and Chun Ching Liu. *Formal analysis*: Amanda Rodriguez Betancourt, Chun Ching Liu, Yi‐Chen Chiang and Hsun‐Liang Chan. *Investigation*: Hsun‐Liang Chan, Amanda Rodriguez Betancourt, Chun Ching Liu, and Patrick R. Schmidlin. *Software*: Amanda Rodriguez Betancourt and Hsun‐Liang Chan. *Writing—original draft and preparation*: Hsun‐Liang Chan, Chun Ching Liu and Patrick R. Schmidlin. Writing review and editing: Hsun‐Liang Chan, Amanda Rodriguez Betancourt, Chun Ching Liu, Yi‐Chen Chiang and Patrick R. Schmidlin.

## CONFLICT OF INTEREST STATEMENT

The authors declare no conflict of interest.

## ETHICS STATEMENT

Ethical approval and consent for participation were obtained for previous studies.

## Supporting information

Video 1. In vivo tissue perfusion assessment with color flow (color velocity) ultrasound in a peri‐implantitis case. Red and blue hue pixels are superimposed on gray B‐mode pixels, where detected velocities exceed a wall filter setting. In this video, velocities towards the transducer are labeled as positive and depicted in red/yellow, whereas blue/cyan pixels are labeled as negative and away from the transducer and can indicate the severity of inflammation.

Video 2. Intrasurgical implant surface decontamination on a peri‐implantitis case under the operating microscope ( ~ 30X). Powered scaler was used to cavitate the calcified tissues firmly attached to the implant.

Video 3. Membrane fixation with pin/screws to enhance wound stability in the reconstructive procedure in high magnification under the operating microscope.

## Data Availability

The data used to support the findings of the study can be obtained from the corresponding author upon request.
